# Coronavirus-like Core–Shell-Structured Co@C for Hydrogen Evolution via Hydrolysis of Sodium Borohydride

**DOI:** 10.3390/molecules28031440

**Published:** 2023-02-02

**Authors:** Shuyi Su, Kailei Chen, Xu Yang, Dai Dang

**Affiliations:** 1 School of Chemical Engineering and Light Industry, Guangdong University of Technology, Guangzhou 510006, China; 2 Jieyang Branch of Chemistry and Chemical Engineering, Guangdong Laboratory (Rongjiang Laboratory), Jieyang 515200, China

**Keywords:** cobalt catalysts, sodium borohydride, hydrogen evolution, core–shell, metal–organic-framework

## Abstract

Constructing a reliable and robust cobalt-based catalyst for hydrogen evolution via hydrolysis of sodium borohydride is appealing but challenging due to the deactivation caused by the metal leaching and re-oxidization of metallic cobalt. A unique core–shell-structured coronavirus-like Co@C microsphere was prepared via pyrolysis of Co-MOF. This special Co@C had a microporous carbon coating to retain the reduced state of cobalt and resist the metal leaching. Furthermore, several nano-bumps grown discretely on the surface afforded enriched active centers. Applied in the pyrolysis of NaBH_4_, the Co@C-650, carbonized at 650 °C, exhibited the best activity and reliable recyclability. This comparable performance is ascribed to the increased metallic active sites and robust stability.

## 1. Introduction

Hydrogen has particularly appealing merits overwhelming conventional fossil fuels, such as zero carbon emission, high energy density, and being almost inexhaustible on the Earth, which is recognized as one of the most fundamental future alternative energy sources [1,2]. As it is sourced for fuel cell devices, hydrogen generation and storage have been bottlenecks limiting its practical application and need to be resolved urgently [3]. Of these numerous kinds of protocols for hydrogen generation and storage, hydrolysis of sodium borohydride (NaBH_4_) to yield hydrogen with high purity has served as an efficient route to commercial utilization [4]. The advanced attributes of high hydrogen content and tunable hydrogen release property make it a promising strategy to realize the practical application of hydrogen energy [5].

Assisted by the presence of catalysts, NaBH_4_ can liberate pure gaseous hydrogen by the manner of hydrolysis at mild temperatures (<100 °C) [6,7]. Noble metals, such as Pt [8], Pd [9], and Ru [10], exhibit excellent performance, yet their scarce reservation and rising cost challenge their coming industrial applications. Alternatively, non-precious metal catalysts, such as Ni [11,12] and Co [13,14], have been available to smooth this chemical transformation. For example, metal borides are widely used for these transition-metal-based catalysts such as cobalt or nickel borides [15,16,17,18]. Despite the high hydrogen yield, those metal borides are subject to aggregation during the liquid-phase reaction, causing the unfavorable issue of catalyst deactivation. A similar obstacle is also confronted by the highly dispersed metal nanoparticles catalyst due to their high surface energy. A plausible solution to address this problem is coating the active component with a porous carbon shell, thus allowing better dispersion and resist-aggregating of these metal nanoparticles [19,20]. In addition, the carbon shell could resist well the alkaline medium, which is generally used to impede the undesired spontaneous hydrolysis of NaBH_4_ [21,22].

In the conventional carbon coating techniques, the organic molecules are mixed simultaneously with metal colloids or nanoparticles and transformed into a metal–organic hybrid via hydrothermal treatment [23]. Subsequent pyrolysis gives rise to an amorphous or graphitic carbon coating on the metal compounds [24]. Up until now, the usage of metal–organic frameworks (MOFs) as the parent precursor to access metal-containing carbon materials has been variously reported [25,26,27,28]. Compared with the conventional one, the MOF-derived route can retain the morphology and structure of the parent MOF, allowing facile regulation of the resulting compositions and structures, especially the porosity [29]. As many studies focused on applying various MOFs to acquire carbon-coating metal catalysts with special morphology or micro-structure, the correlation between the structure and resulting activity on these MOF-derived composites toward hydrolysis of NaBH_4_ remains elusive [30].

In this contribution, carbon-coating cobalt nanospheres with coronavirus-like morphology were successfully synthesized by in situ pyrolysis of Co-MOF. The resulting core–shell-structured Co@C nanospheres were evaluated for hydrolysis of NaBH_4_. Benefiting from the advantages brought by the micro-porous carbon shell, resist-oxidized properties, and unique structure of nano-bumps on the surface, the as-synthesized Co@C composite exhibited high catalytic performance for hydrolysis of alkaline sodium borohydride.

## 2. Results and Discussion

### 2.1. Characterization of Co@C

**Figure 1:**
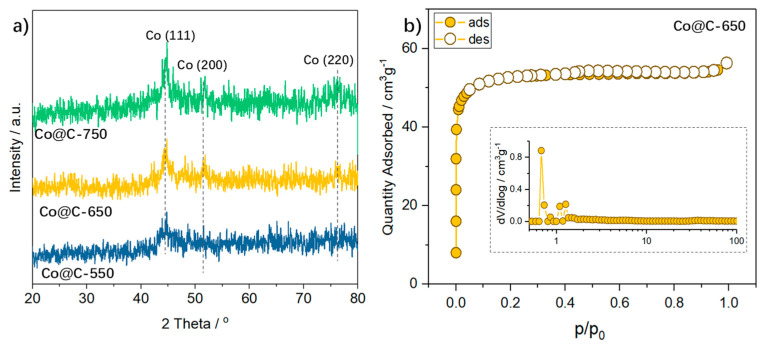
(**a**) XRD pattern of Co@C carbonized with various temperatures, (**b**) N_2_ adsorption/desorption isotherms with pore size distribution (inset) of Co@C−650.

Figure 1a shows the XRD pattern of the MOF-derived Co@C catalysts at different carbonization temperatures. A weak broad peak located at approximately 2θ = 25° belongs to the (002) plane of the graphite–carbon matrix, suggesting the co-presence of the carbon with the Co@C composites. Three well-resolved peaks centered at 44, 52, and 75.8° of 2θ observed for all the Co@C samples are assigned to (111), (200), and (220) crystal planes of cubic cobalt, respectively. The (111) peak becomes more prominent as the pyrolysis temperature is elevated. Using the Scherrer equation, we separately calculated the crystallite size of the Co nanoparticles, which increases from 29 nm to 63 nm for the sample carbonized from 550 to 750 °C.

Nitrogen adsorption–desorption profiles in Figure 1b present the porous structure of the Co@C−650 composites. It exhibits a type-I sorption profile with almost reversible ads-desorption plots, suggesting the presence of micropores within the carbon matrix. The corresponding pore size distribution plot (in Figure 1b) indicates narrowed micropores centered at 0.7 nm for those Co@C−650. The other two samples resemble sorption profiles as that of Co@C−650, which are not shown, for brevity. The specific surface areas, pore sizes, and total pore volumes are summarized in Table 1. 

**Figure 2:**
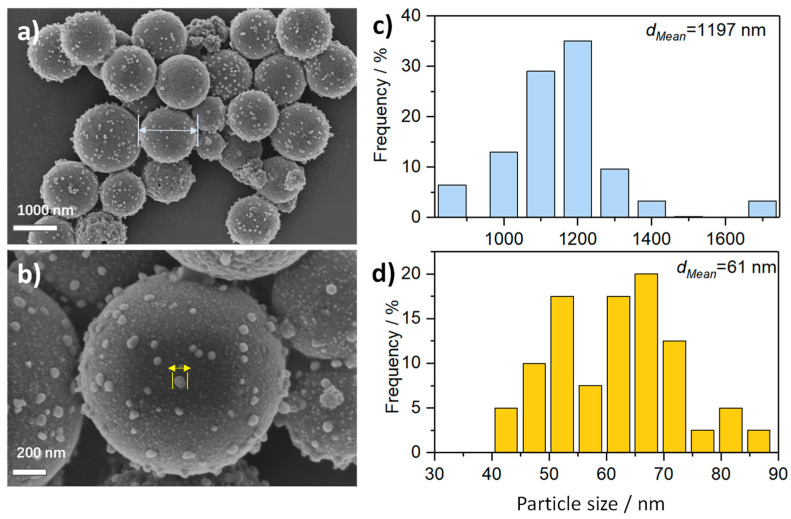
(**a**,**b**) SEM images of Co@C−650. The corresponding particle size distribution plots for (**c**) nano-bumps and (**d**) coronavirus-like microspheres.

The SEM images of Co@C−650 (Figure 2a,b) display a microsphere morphology with a narrowed size distribution of 1–1.4 μm. Interestingly, some discrete bumps are seen on the surface of these spheres, rendering the Co@C−650 like a coronavirus. Such a coronavirus-like morphology is rarely reported in the literature [31,32,33,34]. As illustrated by the magnified image, these bumps are in a size range of 40–90 nm. Compared with the Co-BTC microspheres that have smooth surfaces, these bumps seem to grow in situ from the surface during carbonization. Nevertheless, these bumps decrease and disappear with the increase in carbonization temperature (see Figure S1 of Supporting Information).

**Figure 3:**
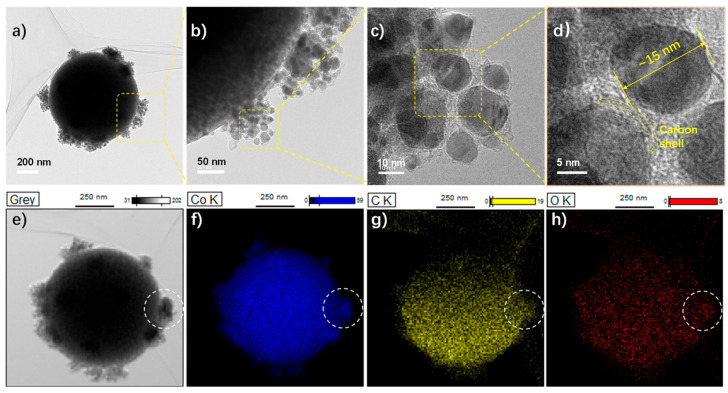
(**a**–**d**) HRTEM images of the Co@C-650 with corresponding (**e**–**h**) element mapping.

HRTEM images are recorded to disclose the micro-morphology and composition of the Co@C−650 composites. These nano-bumps are composed of small cobalt nanoparticles in the range of 10–20 nm (Figure 3a,b). As shown in the selected enlarged region (Figure 3c,d), these microspheres are encapsulated by a graphitic carbon, as indicated by some fringes. The corresponding element mapping in Figure 3e–h verifies the co-presence of cobalt, oxygen, and carbon. Comparing the selected region (indicated by a dotted circle), these nano-bumps particles are carbon-coated cobalt nanoparticles. The presence of oxygen indicates the easy-oxidizable properties of metallic cobalt surfaces. 

**Figure 4:**
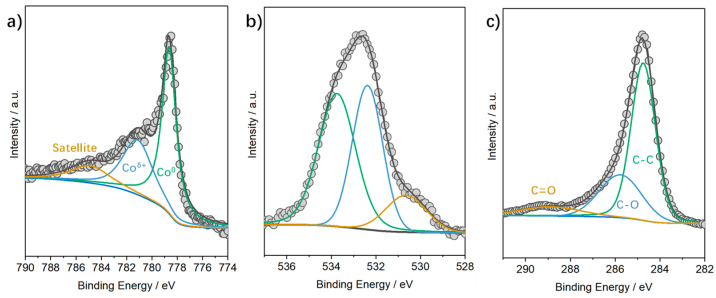
XPS characterization of Co@C−650: (**a**) Co 2p, (**b**) O 1s, and (**c**) C 1s.

XPS characterization was used to access the electronic state of the catalyst. The Co 2p spectra were fitted by three core-level peaks located at 778.6, 781.3, and 785.0 eV (Figure 4a), which were assigned to the metallic Co^0^, oxidized Co^δ+^, and satellite peak, respectively [35]. The metallic cobalt species are likely oxidized; the presence of a carbon shell can somewhat retain the metallic state. Accordingly, the presence of the O 1s peak is related to the partial oxidation of the cobalt on the surface (Figure 4b). The spectra of C 1s were deconvoluted into three peaks at 284.8 eV (C-C), 285.6 eV (C-O), and 288.9 eV (C=O) (Figure 4c), which can be attributed to the C-C bond of sp^3^-hybridized carbon atoms, C-O derived from the adsorbed carbon contaminant (e.g., CO_2_), and C=O bond of the organic residual [19,36], respectively. 

### 2.2. Catalytic Performance

**Figure 5:**
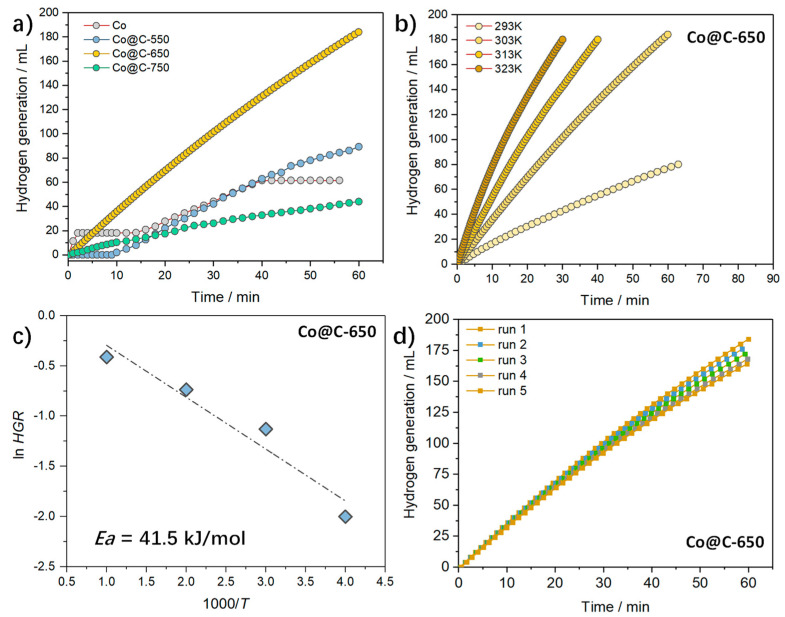
(**a**) Comparative performance toward hydrolysis of NaBH_4_ over various catalysts; (**b**) effect of reaction temperature over the optimized Co@C−650 and (**c**) corresponding Arrhenius plot; (**d**) recycle test for the optimized Co@C−650.

Hydrolysis of NaBH_4_ was utilized to evaluate the catalytic activity of these cobalt catalysts. To stabilize the NaBH_4_, an alkaline medium of 2 wt.% NaOH solution was used, and the final results were calibrated by the blank test. As shown in Figure 5a, the commercial cobalt nanoparticles (Co NPs, Macklin) show a comparable hydrogen evolution rate as the Co@C−550, with some intermittent stagnation. This could be associated with the blocking reaction sites on the Co NPs. These Co@C treated with various carbonization temperatures show a rising, almost straight and smooth line, suggesting their relatively stable hydrogen evolution rate. The Co@C−650 displays the fastest reaction rate, with an HGR of 330 mL min^−1^ g_cat_^−1^. We interestingly found that: (i) the Co@C−750 holds the highest surface area but with the lowest activity; (ii) the Co@C-550 has the smallest crystallite size yet with lower activity than the Co@C−650. Considering the resembling microporosity, we assume that the enhanced activity observed on the Co@C−650 could be ascribed to its exclusive nano-bumps distributed on the microsphere’s surface. These nano-bumps could offer an increased surface area to adsorb these BH^4−^ and OH^−^ to form M-BH_4_ and M-OH species, which are the most abundant reactive intermediates [37]. The hydrolysis mechanisms occurring on the cobalt surface remain elusive, necessitating much exploration of the nature of kinetics, active centers identification, intermediate kinetics, etc., to unveil in the future.

Figure 5b gives the hydrogen evolution as a function of time-on-stream over the Co@C−650 recorded at different reaction temperatures. Expectedly, the Co@C−650 shows an evident temperature dependence, in which the reaction rate rises accordingly with the increasing temperature. We acquired the Arrhenius plot by plotting the lnk versus 1/*T* and obtained an apparent activation energy of 41.5 kJ/mol for the Co@C−650, as shown in Figure 5c, which is low compared to those values obtained in several reports by others [38,39,40].

Finally, the reusability of Co@C−650 was investigated, as is presented in Figure 5d. After five cycles of the run, the HGR decreases by, ca., 10%. Activity decline is commonly reported by scientists in their cobalt-based catalyst [41,42,43].Metal leaching of cobalt due to erosion by the OH^−^ has been recognized as the main cause of the deactivation of the catalysts during the hydrolysis of NaBH_4_ [29]. The spent Co@C−650 was characterized by XRD, nitrogen sorption, ICP, etc. (see Figure S2 of Supporting Information). The crystallite, porosity, and nano-bumps morphology were well-retained, and negligible metal leaching was detected. Hence, we speculate that the surface adsorption of the reactant immediate or other unknown surface reconstruction may be responsible for this limited activity alteration.

## 3. Materials and Methods

### 3.1. Catalyst Preparation

All chemicals were purchased from Macklin, China, and used as received. 

Synthesis of Co-MOF. According to our previously reported hybridization route [44], the synthetic process for the Co-MOF was as follows: first, 0.34 g of cobalt nitrate hydrate (Co(NO_3_)_2_·6H_2_O) and 0.34 g of 1, 3, 5-benzene tricarboxylic acid (BTC) were dissolved in 20 mL of ethanol and stirred for 0.5 h. The mixed solution was subsequently transferred to a 50 mL Teflon-lined autoclave and kept in the oven for 12 h at 150 °C, naturally cooled down. Then, the purple product was washed with ethanol successively after filtration and dried overnight at 60 °C.

Pyrolysis of Co-MOF to access Co@C catalyst. The as-prepared Co-MOF sample was heated to 650 °C with a heating rate of 5 °C min^−1^, calcinated for 2 h under an Ar atmosphere, and then cooled to ambient temperature. Tuning the carbonization temperature, we prepared a series of Co@C and denoted it as Co@C−*T*, where *T* stands for the carbonization temperature. Reference cobalt nanoparticles were purchased commercially from Macklin Cop., Shanghai, China.

### 3.2. Structure Characterizations

X-ray diffraction (XRD) patterns were obtained with a D/Max-IIIA X-ray diffractometer (Rigaku, Tokyo, Japan) using Cu Kα radiation. Scanning electron microscopy (SEM) was performed using an S-4800 FESEM (Hitachi, Tokyo, Japan) to observe morphological and structural analysis. Transmission electron microscopy (TEM) was carried out on a JEM-2010 microscope (JEOL, Tokyo, Japan) using an accelerating voltage of 200 kV. N_2_ adsorption–desorption isotherms were measured with a Tristar 3010 isothermal nitrogen sorption analyzer (Micromeritics, Norcross, GA, USA) using a continuous adsorption procedure. X-ray photoelectron spectroscopy (XPS) was performed with an AXIS Ultra DLD (Kratos, Manchester, UK) to examine the catalysts’ electronic properties. All XPS spectra were calibrated with the C 1s peak at a binding energy of 284.8 eV.

### 3.3. Catalyst Evaluation

The hydrolysis of NaBH_4_ was used to evaluate the catalytic activity of the catalysts. Typically, the catalyst (10 mg) was ultrasonically dispersed in 5 mL of deionized water. Then, the catalyst was quickly added with a mixture of NaOH aqueous solution (2 wt.%) and NaBH_4_ (2 wt.%). The hydrolysis reaction was conducted at 303 K and stirring was continued. The amount of hydrogen generated was determined by a water displacement method. The hydrogen generation rates (HGRs) were reported as (mL_H2_ min^−1^ g_cat_^−1^) [45] and determined using the following equation:

(1) $$\mathrm{Hydrogen}\;\mathrm{generation}\;\mathrm{rate}\;\left(\mathrm{HGR},{\;\mathrm{mL}\;\min}^{-1}{\;g}_{\mathrm{cat}}^{-1}\right)=\frac{V\left(\mathrm{mL}\right)}{t\left(\min\right)\times \mathrm{Wt}.\mathrm{cat}\left(g\right)}$$

where *V* is the volume of water displaced by hydrogen gas in mL, *t* is the time in minutes, and Wt. is the catalyst weight in grams.

## 4. Conclusions

Pyrolysis of Co-MOF generated in situ a unique core–shell-structured Co@C microsphere with a special coronavirus-like morphology. Several nano-bumps grown discretely on the surface offered an enhanced number of active sites for hydrolysis of NaBH_4_. The microporous carbon coating can effectively resist the metal leaching and retain the reduced cobalt state. The Co@C carbonized at 650 °C afforded the highest activity, with a limited activity decline of 10% and low activation energy of 41.5 kJ/mol.

## Figures and Tables

**Table 1:** Sample specifications of various Co@C catalysts.

Catalyst	S_BET_ (m^2^/g)	Pore Volume (cm^3^/g)	Pore Size (nm)	Crystallite Size ^a^ (nm)
Co@C−550	158	0.09	3.4	29
Co@C−650	156	0.08	3.1	46
Co@C−750	189	0.10	3.2	63

^a^ Derived from XRD tests, was calculated using the Debye–Scherrer formula.

## Data Availability

Not applicable.

## Supplementary Materials

The following supporting information can be downloaded at: https://www.mdpi.com/article/10.3390/molecules28031440/s1, Figure S1: SEM image of Co@C-750; Figure S2: XRD pattern and nitrogen sorption with corresponding pore size distribution, and (c) TEM image of the spent Co@C-650.

## Sample Availability

Samples of the compounds are available from the authors.
