# Supplementary material for: Coronavirus-like Core–Shell-Structured Co@C for Hydrogen Evolution via Hydrolysis of Sodium Borohydride
Source: Molecules. 2023 Feb 2;28(3):1440. doi: 10.3390/molecules28031440 (PMC9919990; doi:10.3390/molecules28031440)
Supplement: Supplementary file 1 [file molecules-28-01440-s001.zip › molecules-2174985-supplementary.pdf]

Supporting information

Coronavirus-like core-shell structured Co@C for hydrogen evolution via hydrolysis of sodium borohydride

Shuyi Su [1], Kailei Chen [1,2], Xu Yang [1,2]*, Dai Dang [1,2]*

[1] School of Chemical Engineering and Light Industry, Guangdong University of Technology, Guangzhou, 510006, P. R. China;

[2] Jieyang Branch of Chemistry and Chemical Engineering, Guangdong Laboratory (Rongjiang Laboratory), Jieyang 515200, China;

* Correspondence: yangxu@gdut.edu.cn;

1. Figures

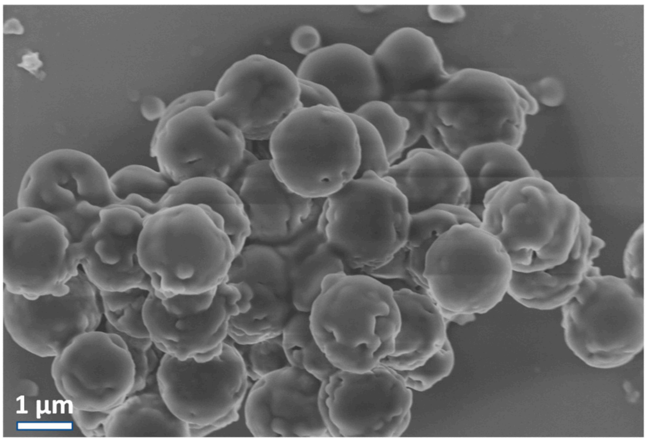

Figure S1 SEM image of Co@C-750. The nano-bumps disappear when the carbonization temperature rises to 750 °C, suggesting an optimized temperature needed for nano-bumps' growth.

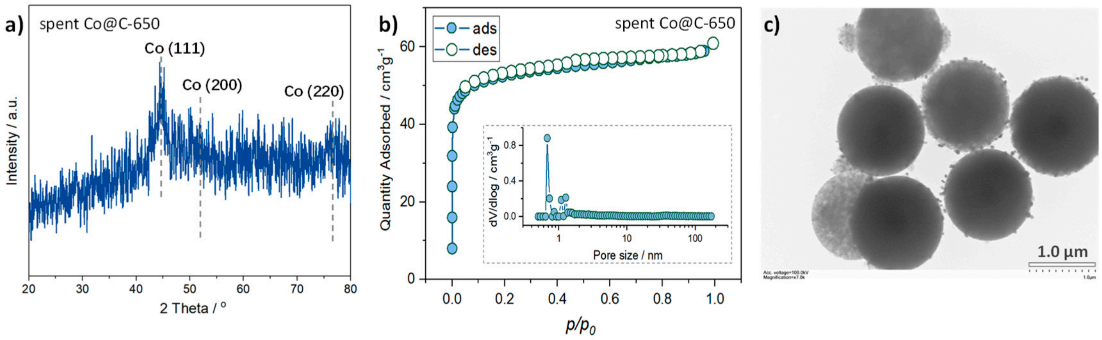

Figure S2 (a) XRD pattern and (b) nitrogen sorption with corresponding pore size distribution (inset), and (c) TEM image of the spent Co@C-650.
